# Supplementary material for: Tunable inertia of chiral magnetic domain walls
Source: Nat Commun. 2016 Nov 24;7:13533. doi: 10.1038/ncomms13533 (PMC5123049; doi:10.1038/ncomms13533)
Supplement: Supplementary Information — Supplementary Figures 1-10, Supplementary Table 1, Supplementary Notes 1-3 and Supplementary References. [file ncomms13533-s1.pdf]

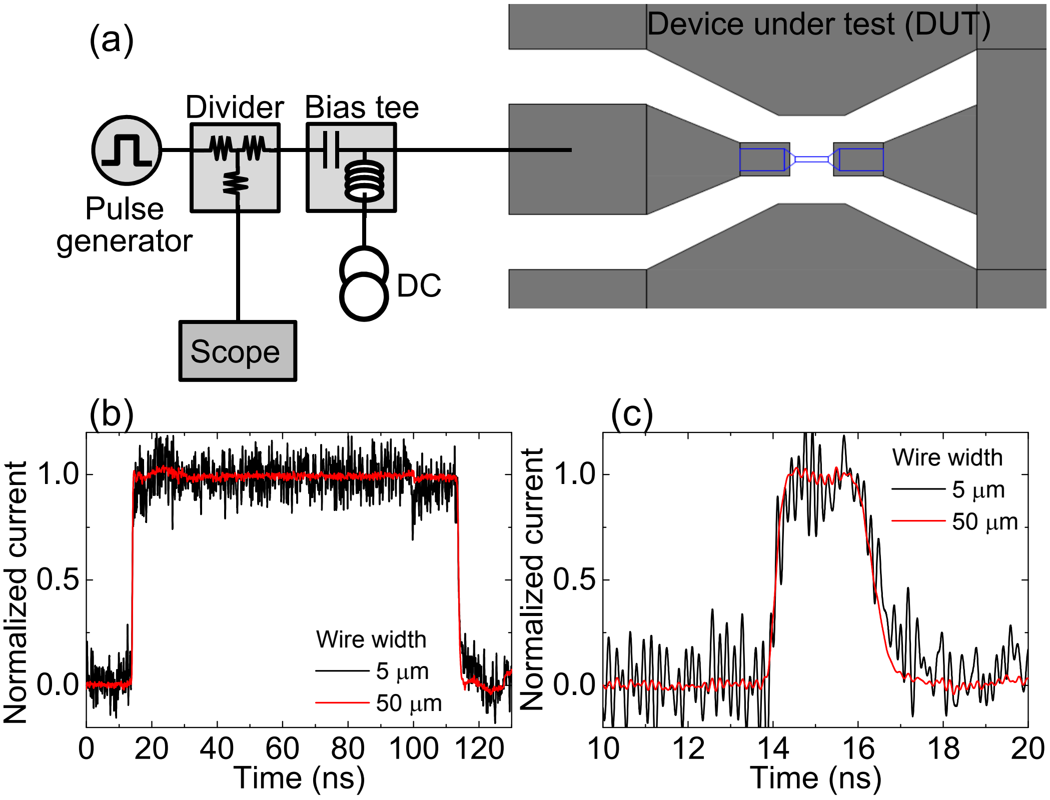

Supplementary Figure 1. Pulse transmission characteristics of a typical device under investigation. (a) Schematic illustration of the measurement setup. (b,c) Measured current that flows into the device when a pulse is applied from the pulse generator for two different devices, *w*~5 μm and ~50 μm. The current is estimated using the time domain reflection measurements. The pulse length is ~100 ns (b) and ~2 ns (c). Results are from film set A.

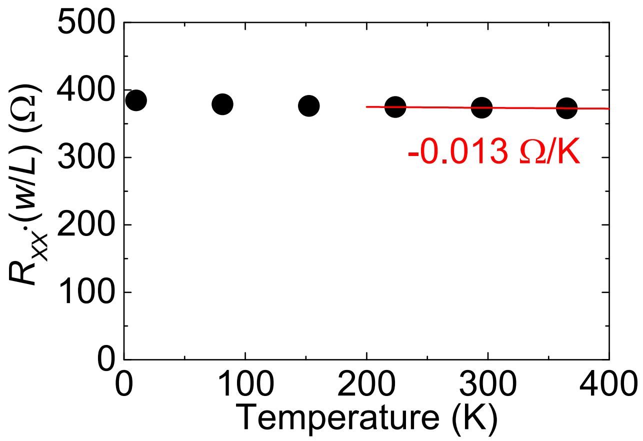

Supplementary Figure 2. Temperature dependence of the device resistance. (a) The resistance (R_{XX}) of a wire made from Sub.|W(~3.1)/$Co_{20}Fe_{60}B_{20}$(1)/MgO(2)/Ta(1) is measured as a function of measurement temperature (T). The resistance is normalized by the length (L) and width (w) of the wire. The resistance hardly changes with temperature: the slope of $R_{XX}\cdot(w/L)$ vs. T is ~−0.013 W/K. Results are from film set B.

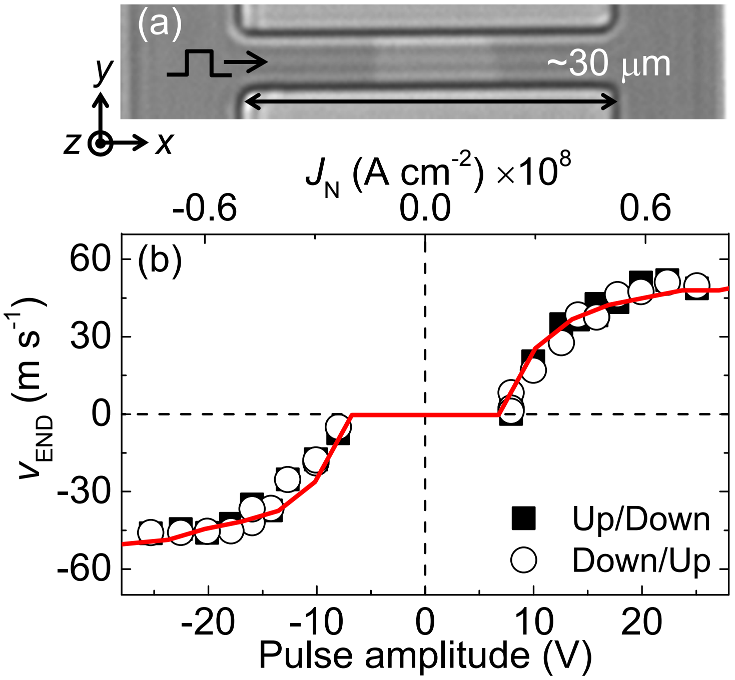

Supplementary Figure 3. Fitting of velocity vs. current density using the 1D model. (a) Optical (Kerr) microscopy image of the device used to study domain wall motion. (b) Pulse amplitude dependence of the quasi-static velocity (v_{END}) for a fixed pulse length ($t_{\mathrm{P}} = 10$ ns). The corresponding current density that flows through the W underlayer is shown in the top axis. The open and solid symbols show the velocity for ↓↑ walls and ↑↓ walls, respectively. Results are from film set B, wire width is ~5 μm. The red solid line represents fitting with the 1D model that takes into account domain wall tilting and pinning. Parameters used: $M_{\mathrm{S}} = 1100$ emu cm^{-3}, $K_{\mathrm{EFF}} = 3.2 \times 10^6$ erg cm^{-3}, $\Delta = \sqrt{A/K_{\mathrm{EFF}}}$ ~6.8 nm ($A = 1.5 \times 10^{-6}$ erg cm^{-1}), $\theta_{\mathrm{SH}} = -0.21$, $D = 0.24$ erg cm^{-2}, $\alpha = 0.05$ and the wire width w=5 μm. A 1D pinning $H_{\mathrm{PIN}}(q) = \frac{1}{2M_{\mathrm{S}} w t_{\mathrm{FM}}} \left(\frac{V_0 \pi}{q_0}\right) sin\left(\frac{\pi}{q_0} q\right)$ that accounts for local imperfections is introduced with V_0=1.6×10^{-11} erg and q_0=7 nm.

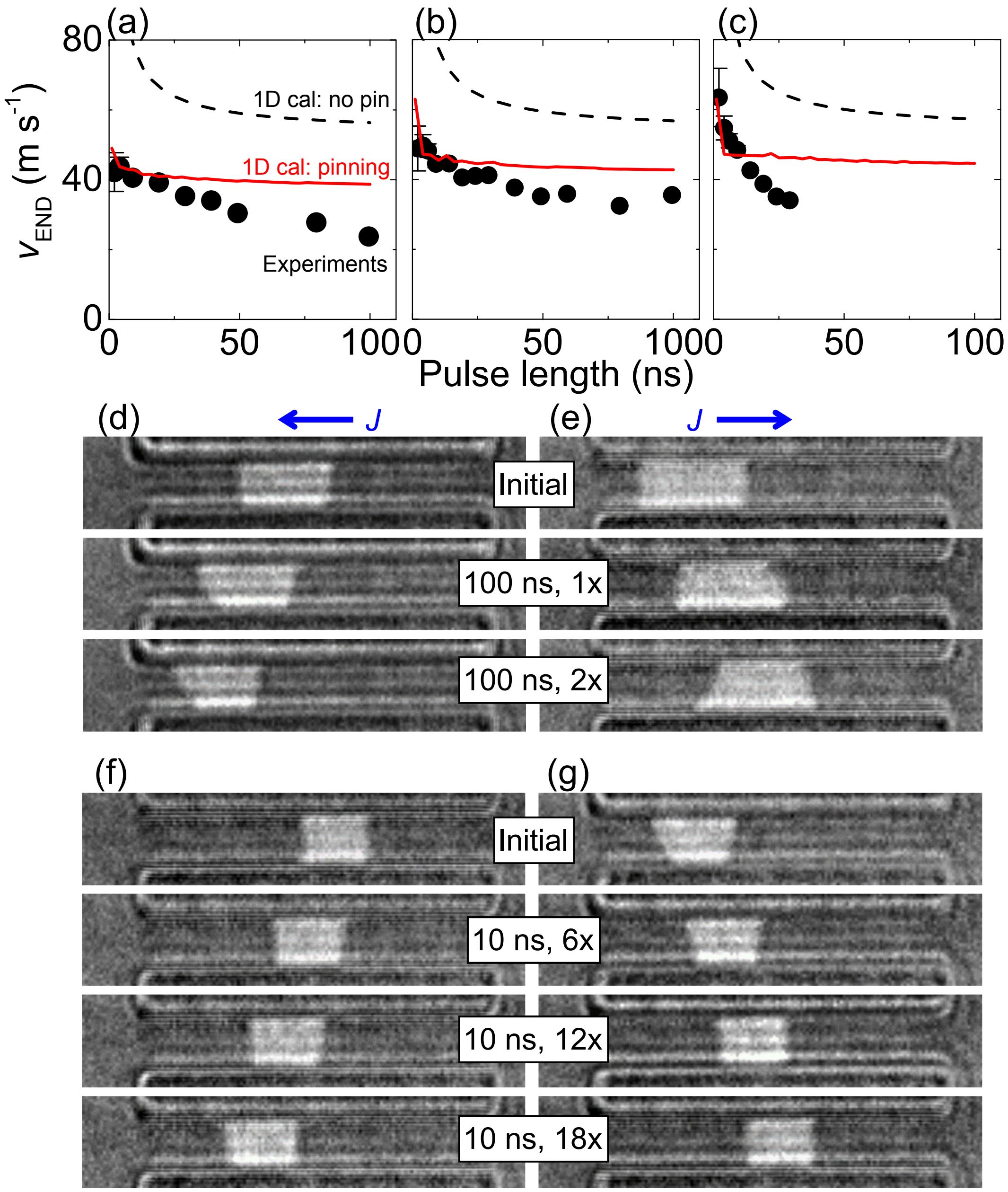

Supplementary Figure 4. Current driven domain wall motion in ~5 μm wide wires. (a-c) Experimentally measured quasi-static velocity (v_{END}, black circles) as a function of pulse length for three different pulse amplitudes: (a) 16 V (J~0.5×10^{8} A cm^{-2}) (b) 20 V (J ~0.6×10^{8} A cm^{-2}) and (c) 25 V (J ~0.8×10^{8} A cm^{-2}). The errors bars represent variation in v_{END} due to the uncertainty in the pulse length. Experimental results are the same with those shown in Fig. 5(a-c). The red solid and black dashed lines show v_{END} calculated using the 1D model with and without pinning, respectively. Parameters used are the same with those shown in Fig. S3 caption. (d-g) Sequences of Kerr images showing the successive wall motion after application of current pulses described in the legend: (d) t_{P} = 100 ns, J<0, (e) t_{P} = 100 ns, J>0, (f) t_{P} = 10 ns, J<0 and (g) t_{P} = 10 ns, J>0. All results are from film set B, wire width is ~5 μm.

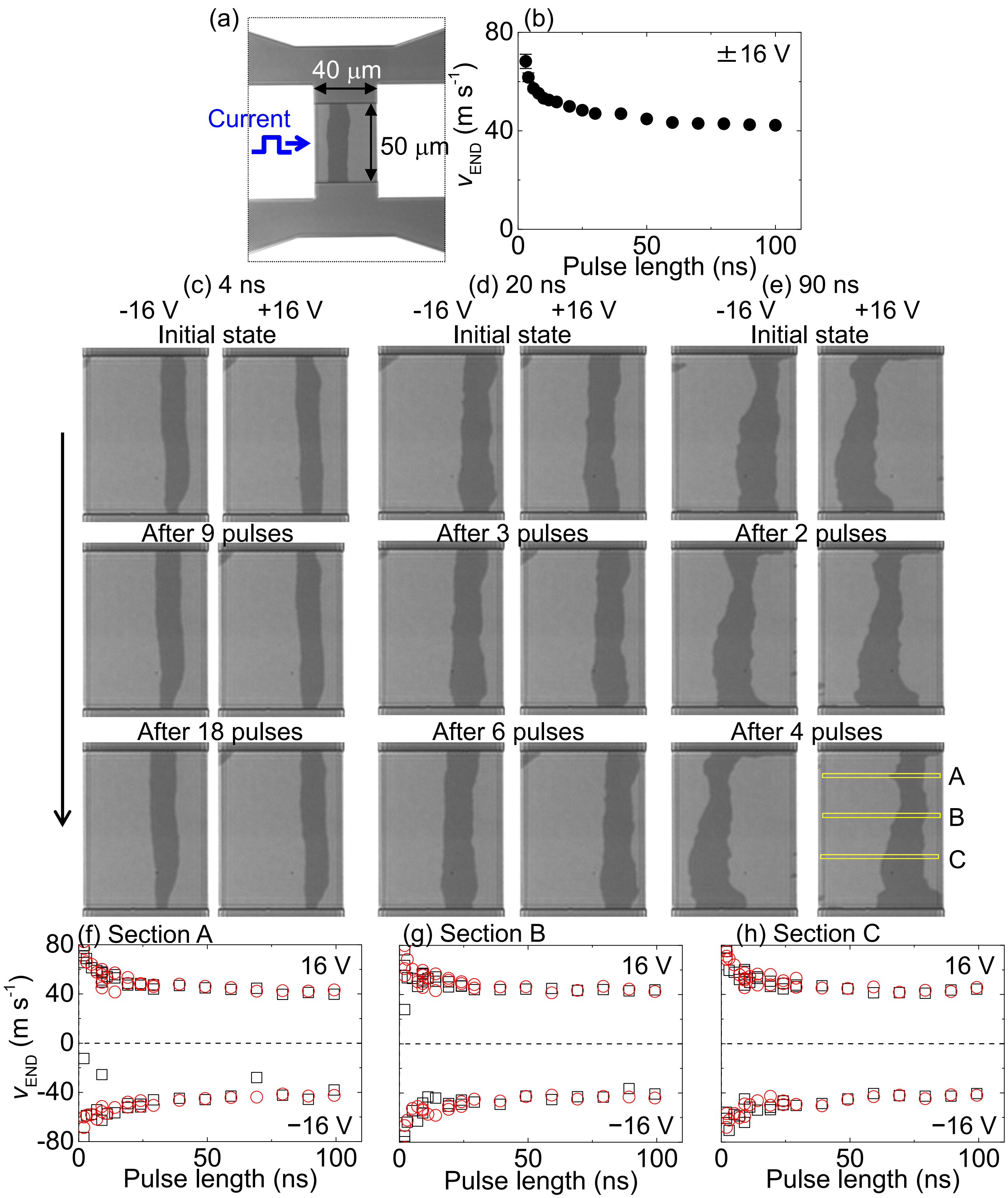

Supplementary Figure 5. **Current driven domain wall motion in ~50 μm wide wires.** (a) Representative Kerr microscopy image of the ~50 μm wide wire used to study domain wall motion. (b) Average of the quasi-static velocity (v_{END}) obtained from the three sections A, B, C denoted by the yellow rectangles in (e). The error bars represent standard deviation of the velocity estimated in the three sections. (c-e) Sequences of Kerr images showing the successive wall motion after application of current pulses described in the legend. The left and right panels show images when -16 V and +16 V pulses are applied, respectively. The pulse length is (c) 4 ns, (d) 20 ns and (e) 90 ns. (f-h) v_{END} as a function of pulse length obtained from the three sections A (f), B (g) and C (h) denoted by the yellow rectangles in (e). The pulse amplitude is fixed to ±16 V. Black squares and red circles represent v_{END} for ↑↓ and ↓↑ walls. The film structure is Sub.|W(~3.6)/$Co_{20}Fe_{60}B_{20}$(1)/MgO(2)/Ta(1). Results are from film set A, wire width is ~50 μm.

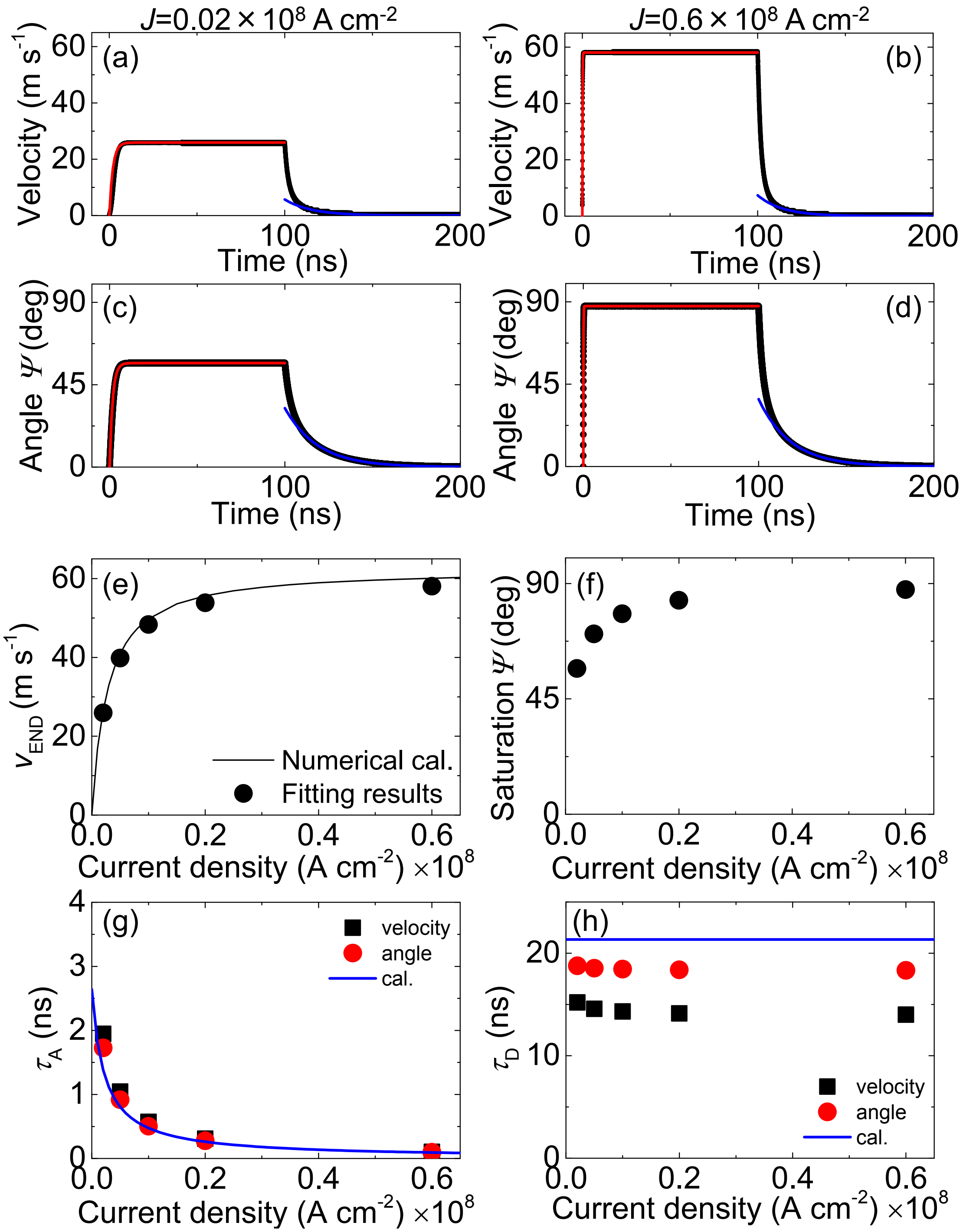

Supplementary Figure 6. Analyses of the linearized 1D model. (a-d) Instantaneous DW velocity $v(t)$ (a,b) and the wall magnetization angle $\psi(t)$ (c,d) for a fixed current density of $J = 0.02 \times 10^8$ A cm^{-2} (a,c) and $J = 0.6 \times 10^8$ A cm^{-2} (b,d) flowing through the heavy metal layer. The current pulse length is (t_P) is 100 ns. Fit to data in appropriate ranges using Eq. (S6) are shown by the red and blue solid lines. (e-h) Current density J dependence of saturation velocity (e), the equilibrium wall angle (f), the acceleration time (g) and the deceleration time (h) when the current is turned on. Results are obtained by the fitting process described in (a-d). The blue solid line in (g) and (h) are the analytical solutions provided in Eqs. (2) and (3), respectively. Parameters used: $M_S = 1100$ emu cm^{-3}, $K_{EFF} = 3.0 \times 10^6$ erg cm^{-3}, $\Delta = \sqrt{A/K_{EFF}}$ ~7.0 nm ($A = 1.5 \times 10^{-6}$ erg cm^{-1}), $\theta_{SH} = -0.21$, $D = 0.24$ erg cm^{-2}, $\alpha = 0.05$ and w=5 μm.

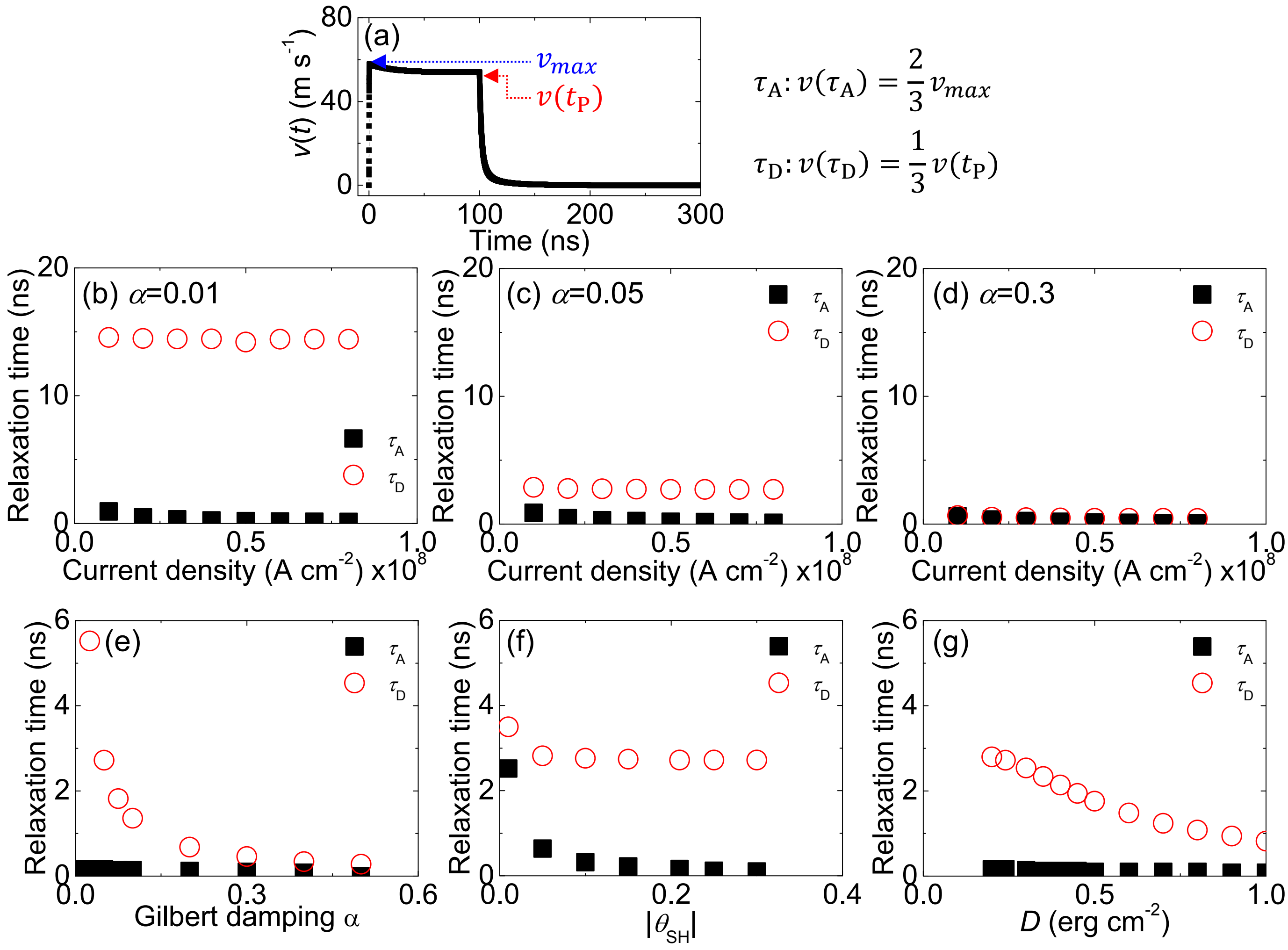

Supplementary Figure 7. Numerical calculations of the relaxation times using the 1D model. (a) Instantaneous DW velocity $v(t)$ when a current density of $J = 0.6 \times 10^8$ A cm^{-2} is flowing through the heavy metal layer. The current pulse length is (t_P) is 100 ns. The maximum velocity (v_{max}) and the velocity at the end of the pulse, i.e. the terminal velocity ($v(t_P)$) are illustrated schematically. (b-g) Acceleration (τ_A) and deceleration (τ_D) times numerically computed using the definition described in (a). (b-d) τ_A and τ_D are plotted as a function of current density (b-d), Gilbert damping constant α (e), spin Hall angle θ_{SH} (f) and the DM exchange constant D (g). The Gilbert damping constant α is varied for the plots shown in (b-d): α =0.01 (b), 0.05 (c) and 0.3 (d). The parameters used unless specified are: $M_S = 1100$ emu cm^{-3}, $K_{EFF} = 3.2 \times 10^6$ erg cm^{-3}, $\Delta = \sqrt{A/K_{EFF}} \sim 6.8$ nm ($A = 1.5 \times 10^{-6}$ erg cm^{-1}), $\theta_{SH} = -0.21$, $D = 0.24$ erg cm^{-2}, $\alpha = 0.05$ and w=5 μm.

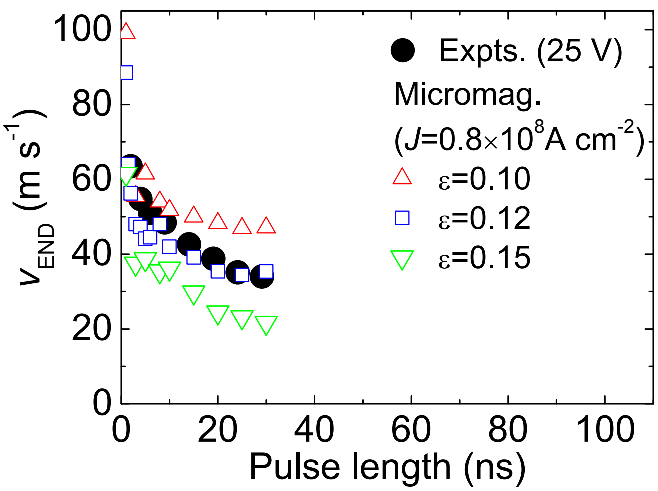

Supplementary Figure 8. **Micromagnetic simulations of the quasi-static velocity: grain pattern dependence.** Quasi-static velocity (v_{END}) as function of the pulse length under current pulses of fixed amplitude $J = 0.8 \times 10^8$ A cm^{-2}. Experimental results (solid circles, same with those shown in Fig. 5(c)) are compared to the full micromagnetic predictions considering different degrees of disorder and grain pattern A. The width of the wire (w) is ~5 μm for the experiments (film set B) and w is set to 1.5 μm for the simulations.

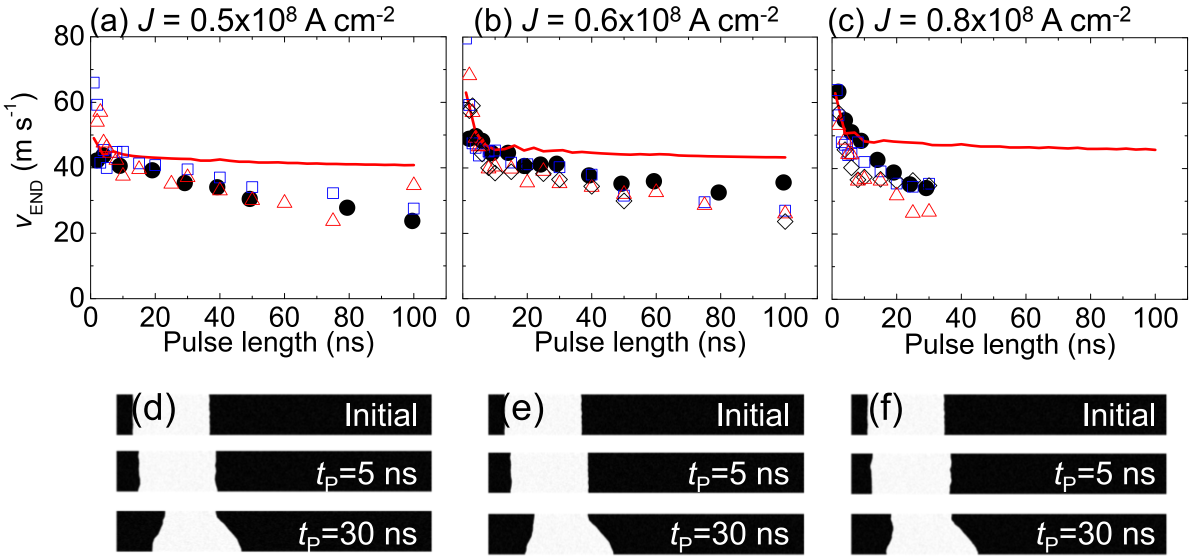

Supplementary Figure 9. Comparison of calculated quasi-static velocity using 1D model and micromagnetic simulations. (a-c) Quasi-static velocity (v_{END}) as function of the pulse length under current pulses of fixed amplitude: (a) $J = 0.5 \times 10^8$A cm^{-2}, (b) $J = 0.6 \times 10^8$A cm^{-2} and (c) $J = 0.8 \times 10^8$A cm^{-2}. Experimental results (black circles, same with those shown in Fig. 5(a-c)) are compared to full micromagnetic simulations (open symbols). The degree of disorder in the micromagnetic simulations is fixed to $\varepsilon = 0.12$. Blue, red and black open symbols correspond to micromagnetic results using the following conditions: blue squares are for grain pattern A with T=0 K, red triangles are for grain pattern A with T=300 K and black diamonds are for grain pattern B with T=0 K. Grain patterns A and B are randomly generated. (d-f) Micromagnetic snapshots of the initial state and states long after application of current pulses (t_{P} = 5 and 30 ns). The width of the wire (w) is ~5 μm for the experiments (film set B) and w is set to 1.5 μm for the simulations.

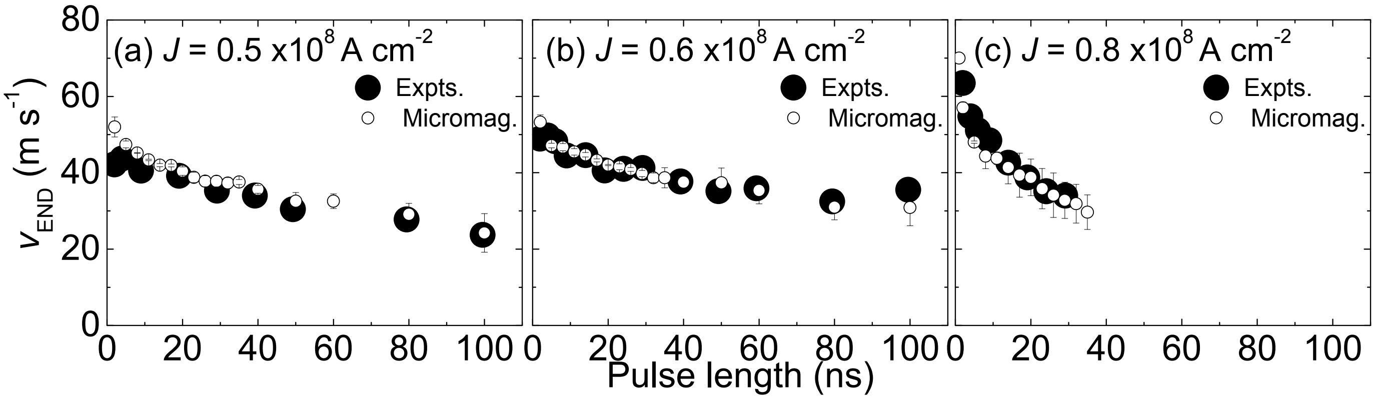

Supplementary Figure 10. **Micromagnetic simulations of the quasi-static velocity: current density dependence** Quasi-static velocity (v_{END}) as function of the pulse length under current pulses of fixed amplitude. Experimental results (big solid circles, same with those shown in Fig. 5(a-c)) are compared to full micromagnetic simulations (small open circles) with wire width set close to the experiments. (a) $J = 0.5 \times 10^8 \mathrm{A\ cm^{-2}}$, (b) $J = 0.6 \times 10^8 \mathrm{A\ cm^{-2}}$ and (c) $J = 0.8 \times 10^8 \mathrm{A\ cm^{-2}}$. The degree of disorder in the micromagnetic simulations is fixed to $\varepsilon = 0.12$ for grain pattern A with *T*=300 K. A grain size of 20 nm was considered for these micromagnetic results. The width of the wire (*w*) is 5 μm for both the experiments (film set B) and the simulations. The error bars of the simulations represent five independent calculations using different thermal noise patterns.

Supplementary Table 1. Magnetic and transport properties of film sets A and B. The film structure is Sub./W(*d*)/$Co_{20}Fe_{60}B_{20}$(1)/MgO(2)/Ta(1) (units in nanometers). Resistivity (ρ_N), absolute value of the spin Hall angle ($|\theta_{SH}|$), spin diffusion length (λ_N), saturation magnetization (M_S) and the effective magnetic anisotropy energy (K_{EFF}) are listed. For M_S and K_{EFF} values are taken from films with the W layer thickness *d*~3 nm.

Films	ρ_N μΩcm	$\lvert\theta_{SH}\rvert$	λ_N nm	M_S [(a)] emu cm^{-3}	K_{EFF} [(a)] erg cm^{-3}
Set A	150	0.24	1.1	790	2.9×10^6
Set B	125	0.23	1.3	780	3.2×10^6

[(a)] Values when *d*~3 nm

Supplementary Note 1

Experimental setup

Shape of the current pulse

Since short voltage pulses (a few nanoseconds long) are used, any glitches in the transmission line can distort the pulse shape. We thus use time domain reflection measurements to study the temporal evolution of the current that flows into the wire. Supplementary Fig. 1(a) shows schematic illustration of the measurement setup. A constant amplitude voltage pulse is applied from the pulse generator and we measure the reflected voltage pulse, via a power divider, using a real time oscilloscope. The normalized current pulses measured for pulse lengths of ~100 ns and ~2.1 ns are shown in Supplementary Fig. 1(b) and 1(c), respectively. As evident, there is no obvious glitch in the current pulse shape for both pulses. Since the input impedance of the pulse generator is not perfectly 50 Ohm, we take this into account to calculate the current applied to the device. The fluctuations in the signals found at times of ~30 ns and ~100 ns in Supplementary Fig. 1(b) are due to this correction. The difference in the rise and fall times of the pulse (Fig. S1(c)) is largely to do with the pulse generator: the rise and fall times of the pulse generator is <0.3 ns and 0.75 ns, respectively. The pulse length is measured using a real time oscilloscope.

Effect of Joule heating

The device temperature evolution with the current pulse due to Joule heating in a similar structure was reported previously in Ref. 1. The temperature variation was analyzed from anomalous hall resistance. Based on these results, the temperature rise due to Joule heating is expected to be at most ~100 K for the maximum pulse amplitude and length applied to the device in this work. The increase of temperature is smaller for shorter current pulses. The

effect of temperature on the pulse length dependent velocity is evaluated using micromagnetic simulations (see Supplementary Note 3, below).

Since the pulse generator outputs a constant-amplitude voltage pulse, the time evolution of the current that flows into the device may vary if Joule heating takes place. Fortunately, for the heterostructure studied here (W/CoFeB/MgO), the temperature variation of the device resistance is nearly constant due to the amorphous-like structure[2] of the conducting path (CoFeB and W). Supplementary Fig. 2 shows the measurement temperature dependence of the longitudinal resistance (R_{XX}) normalized by the ratio of wire width (w) and wire length (L). Unlike typical metals, the temperature variation of $R_{XX}/(w/L)$ is flat: the slope is ~−0.013 Ω/K. Thus the shape of the current pulse will not be distorted if Joule heating was to occur.

Note that the wall velocity is almost constant with the current density once it saturates; see Fig. 1(a-f) and Supplementary Fig. 3(b). Such saturation of velocity at high current is in accordance with the 1D model (Eq. (1)), which assumes constant magnetic properties at all currents. We thus consider Joule heating at high current has little impact on the magnetic properties of the films.

Domain wall tilting in 5 μm and 50 μm wide wires

As described in the main text, the degree of wall tilting depends on the wire width. In the following, we show results of current induced domain wall tilting and its influence on the velocity in ~5 μm and ~50 μm wide wires.

An optical microscopy image of the ~5 μm wide wire is shown in Supplementary Fig. 3(a). Supplementary Fig. 3(b), circles and squares, show measured v_{END} as a function of pulse amplitude for a fixed pulse length ($t_{\mathrm{P}} = 10$ ns). The corresponding pulse length (t_{P}) dependence of v_{END} is plotted in Supplementary Fig. 4(a-c), circles, for different pulse amplitudes. (The results are the same with those shown in Fig. 5(a-c).) In all cases, the

velocity increases with decreasing pulse length. Signatures of domain nucleation are found for t_P>30 ns when the pulse amplitude is set to ~25 V (Supplementary Fig. 4(c)), which hinders accurate evaluation of the velocity in this regime. In contrast to the pulse length dependent v_END found for the wider wires (~50 μm wide, Fig. 1(g-l)), $v_\mathrm{END}$of the narrower wires (~5 μm wide, Supplementary Fig. 4(a-c)) continues to decrease as the pulse length is increased beyond ~20 ns.

Supplementary Fig. 4(d-g) show sequences of Kerr images when voltage pulses (~16 V) are applied to the ~5 μm wide wire. The bright and dark contrasts correspond to magnetization pointing along +*z* and –*z*, respectively. The top image shows the initial state of the wire in which two domain walls with opposite wall types (↓↑ and ↑↓ walls) are placed. For t_P =100 ns, the domain wall becomes tilted as it moves along the wire. We define the wall tilt angle χ as the angle between the wall normal and +*x* (see Fig. 2(a)). The tilt angle is opposite for the ↓↑ and ↑↓ domain walls and it reverses when the current direction is changed. The way the domain wall tilts is opposite to that if the Oersted field was to tilt the wall. This is in agreement with previous reports, which attribute the DMI as the source of the wall tilting[3-6]. For shorter pulses, the tilting is not obvious from the images.

As discussed in the main text, the wall tilting can influence the wall velocity, in particular, for longer pulses. According to Supplementary Eq. (9), the time it takes for the wall tilting to develop, defined as τ_χ, scales with the square of the wire width. Thus an increase in the wire width by a factor of ten will increase τ_χ by 100. For typical material parameters found in this system, we expect negligible tilting when we increase the width from ~5 μm to ~50 μm for the maximum pulse length (~100 ns) used in the experiments.

To study the wire width dependence of the wall tilting, Supplementary Fig. 5 summarizes the wall motion in ~50 μm wide wire in comparison to that shown in Supplementary Figs. 4.

and 5(a) shows a Kerr microscopy image of a typical ~50 μm wide wire. Successive Kerr images of the magnetic state of the wide wires after application of current pulses are shown in Supplementary Figs. 5(c-e) for 4 ns, 20 ns and 90 ns long pulses (the pulse amplitude is fixed to ±16 V). We do not find observable wall tilting in these wide wires, in contrast to the 5 μm wide wires. However, domain walls tend to be more distorted when longer pulses (~90 ns) are used, Supplementary Fig. 5(e).

To estimate v_{END}, here we divide the wire into small sections and calculate the velocity of wall segments present within each section and take the average of all sections (as described in the Methods section of the main text). The yellow rectangles depicted in Supplementary Fig. 5(e), bottom right panel, show examples of the small sections. The pulse length dependence of v_{END} of the wall segments present in sections A, B and C are displayed in Supplementary Figs. 5(f), 5(g) and 5(h), respectively. The black square and red circles indicate v_{END} for ↑↓ and ↓↑ walls, respectively. The pulse length dependence of v_{END} is similar for all segments of the walls despite the different pinning profile each segment will experience when moving along each section. The velocity obtained from the three sections are averaged and shown in Supplementary Fig. 5(b). As observed for the ~5 μm wide wires (Fig. 5 and Supplementary Fig. 4), the velocity increases for shorter pulses ($t_{\mathrm{P}} \lesssim 20$ ns). However, the gradual reduction in v_{END} at longer pulses is not evident for the ~50 μm wide wires. These results indicate that the tilting effect is small for the ~50 μm wide wires, suggesting that the increase in the velocity for shorter pulses is likely to do with the inertia effect.

Supplementary Note 2

The one dimension (1D) collective coordinate model of a domain wall

Model description

In order to describe the experimental observations, first the dynamics of chiral domain walls under current pulses is studied using the 1D collective coordinate model with the spin Hall torque and the DMI included[1,7-9]. The domain wall dynamics is described using the following three time (t) dependent variables[4,10]: the wall position $q(t)$, the wall magnetization angle $\psi(t)$ and the wall tilting angle $\chi(t)$ ($\psi(t)$ and $\chi(t)$ are defined with respect to +x, see inset of Fig. 3(a)). The tilting of the wall arises due to the DMI. Details of the 1D model used here can be found in Ref. 5.

$$(1+\alpha^2)\frac{\cos\chi}{\Delta}\frac{dq}{dt} = \left[-\frac{\gamma H_{\mathrm{K}}}{2}\sin 2(\psi-\chi) + \Gamma\frac{\pi}{2}\gamma H_{\mathrm{DM}}\sin(\psi-\chi)\right] + \alpha\left[\gamma H_{\mathrm{PIN}}(q) + \Gamma\frac{\pi}{2}\gamma H_{\mathrm{SH}}\cos\psi\right] \quad (1)$$

$$(1+\alpha^2)\frac{d\psi}{dt} = -\alpha\left[-\frac{\gamma H_{\mathrm{K}}}{2}\sin 2(\psi-\chi) + \frac{\pi}{2}\Gamma\gamma H_{\mathrm{DM}}\sin(\psi-\chi)\right] + \left[\gamma H_{\mathrm{PIN}}(q) + \Gamma\frac{\pi}{2}\gamma H_{\mathrm{SH}}\cos\psi\right] \quad (2)$$

$$\alpha\frac{\pi^2}{12\gamma}\left[\tan^2\chi + \left(\frac{w}{\pi\Delta\cos\chi}\right)^2\right]\frac{d\chi}{dt} = -\frac{H_{\mathrm{K}}}{2}\sin(2(\psi-\chi)) + \Gamma\frac{\pi}{2}H_{\mathrm{DM}}\sin(\psi-\chi) - \left[\frac{2K_{\mathrm{EFF}}}{M_S} - \Gamma\frac{\pi}{2}H_{\mathrm{DM}}\cos(\psi-\chi) + H_{\mathrm{K}}\cos^2(\psi-\chi)\right]\tan\chi \quad (3)$$

For out of plane magnetized systems, the domain wall magnetization is pointing along the film plane: $\psi = 0, \pi$ and $\psi = \frac{\pi}{2}, -\frac{\pi}{2}$ corresponds to Neel and Bloch walls, respectively. The magneto-static anisotropy field associated with the wall is expressed as $H_{\mathrm{K}} = \frac{4t_{\mathrm{FM}}M_{\mathrm{S}}log(2)}{\Delta}$ [5,11], where M_{S} is the saturation magnetization, Δ is the domain wall width parameter (the physical domain wall width is $\pi\Delta$) and t_{FM} is the thickness of the magnetic

layer. α and K_{EFF} are the Gilbert damping parameter and the effective magnetic anisotropy energy of the magnetic layer, respectively. w is the width of the wire. Γ represents the domain wall pattern; $\Gamma = +1$ for the ↑↓ wall and $\Gamma = -1$ for the ↓↑ wall. $H_{\mathrm{PIN}}(q)$, H_{DM} and H_{SH} are the pinning field, the Dzyalonshinskii-Moriya (DM) offset field and the spin Hall effective field, respectively. H_{DM} and H_{SH} can be explicitly written as $H_{\mathrm{DM}} = \frac{D}{\Delta M_{\mathrm{S}}}\Gamma$ and $H_{\mathrm{SH}} = -\frac{\hbar\theta_{\mathrm{SH}}}{2eM_{\mathrm{S}}t_{\mathrm{FM}}}J$[12,13], where D is the DM exchange constant, θ_{SH} is the spin Hall angle of the heavy metal layer and J is the current density that flows into the heavy metal layer. Here, only the damping-like component of the spin Hall torque[13] is included. For simplicity, the spin transfer torques, both the adiabatic and the non-adiabatic terms[14], that occur within the magnetic layer is neglected since their contribution is much smaller than that of the damping-like spin Hall torque for the system under consideration[1]. The definitions of the constants used here are: γ is the Gyromagnetic ratio, $\hbar$ is the reduced Planck constant and e is the electron charge.

One can linearize Supplementary Eqs. (1) and (2) to obtain the characteristic equation of a domain wall (the wall tilting is set zero here)[1,8,9,15].

$$m\frac{\partial^2 q}{\partial t^2} + \frac{m}{\tau}\frac{\partial q}{\partial t} = F, \tag{4}$$

where m is the effective domain wall mass, τ is the relaxation time and F is the driving force. These parameters are derived as:

$$m = \left(\frac{2M_{\mathrm{S}}}{\gamma}\right)^2 \frac{(1+\alpha^2)wt_{\mathrm{FM}}}{f(\psi_{\mathrm{eq}})} \tag{5}$$

$$\tau = \frac{\Delta}{\alpha}\left(\frac{2M_{\mathrm{S}}}{\gamma}\right)\frac{(1+\alpha^2)}{f(\psi_{\mathrm{eq}})} \tag{6}$$

$$F = \frac{1}{f(\psi_{\rm eq})}\left\{\begin{matrix}\left[-\frac{\partial\sigma}{\partial q}+\left(\frac{2M_{\rm S}}{\gamma}\right)^2\frac{\beta u}{\Delta}\right]\cdot\left.\frac{\partial^2\sigma}{\partial\psi^2}\right|_{eq}+\Gamma\frac{\pi}{2}\gamma\sin\psi_{\rm eq}\left[-\left(\frac{2M_{\rm S}}{\gamma}\right)^2H_{\rm SH}u+\left(\frac{2M_{\rm S}}{\gamma}\right)H_{\rm SH}\left.\frac{\partial\sigma}{\partial\psi}\right|_{\rm eq}\right] \\ +\Gamma\frac{\pi}{2}\gamma\cos\psi_{\rm eq}\left[-\left(\frac{2M_{\rm S}}{\gamma}\right)H_{\rm SH}\left.\frac{\partial^2\sigma}{\partial\psi^2}\right|_{\rm eq}\right]\end{matrix}\right\} \quad (7)$$

Here $f(\psi_{\rm eq})=\left[\left.\frac{\partial^2\sigma}{\partial\psi^2}\right|_{\rm eq}+\Gamma\frac{\pi}{2}\frac{\Delta}{\alpha}\left(\frac{2M_{\rm S}}{\gamma}\right)\gamma H_{\rm SH} sin\psi_{\rm eq}\right]$ and $\psi_{\rm eq}$ is the equilibrium (steady state) magnetization angle of the wall. The domain wall energy density (σ) can be defined as:

$$\sigma = \sigma_0 + M_{\rm S}H_{\rm K}\Delta\cos^2\psi - \pi\Delta M_{\rm S}H_{\rm DM}\cos\psi \quad (8)$$

where σ_0 is the domain wall energy density that is just a constant (i.e., not a function of q or $\psi_{\rm eq}$). Note that $\frac{m}{\tau}=\frac{\alpha w t_{\rm FM}}{\Delta}\left(\frac{2M_{\rm S}}{\gamma}\right)$ gives the friction against the wall motion.

The spin Hall torque tends to rotate the wall magnetization away from the Neel configuration ($\psi_{\rm eq}$~0 or π) to that of the Bloch configuration ($\psi_{\rm eq}\sim\frac{\pi}{2}$ or $-\frac{\pi}{2}$). When current is applied, one can substitute $\psi_{\rm eq}\sim\frac{\pi}{2}$ or $-\frac{\pi}{2}$ into Supplementary Eq. (6) to obtain the acceleration time ($\tau_{\rm A}$), as shown in Eq. (2) of the main text. The deceleration time ($\tau_{\rm D}$) (Eq. (3) of the main text) can be evaluated by substituting $\psi_{\rm eq}$~0 or π in Supplementary Eq. (6).

One can associate the relaxation time τ with the effective wall mass m using the relation that derives from Supplementary Eqs. (5-7), i.e. $m_{\rm A(D)}=\frac{2M_{\rm S}\alpha w t_{\rm FM}}{\gamma\Delta}\tau_{\rm A(D)}$. $m_{\rm A}$ represents the effective mass when the domain wall is driven by current whereas $m_{\rm D}$ corresponds to the effective mass when the wall is at rest (i.e. when the current is turned off). Since the proportionality factor that relates m and τ is a constant, these equations indicate that the effective wall mass is different when the domain wall is driven by current and when it is at

rest. Note that $\tau_{\mathrm{A(D)}}$ and $m_{\mathrm{A(D)}}$ evolve during the transient process and therefore are not constant.

For domain wall tilting, the 1D model predicts that the time it takes to reach the steady state tilting angle (τ_χ) depends on the wire width and the damping constant. According to Boulle *et al.*[4], τ_χ is expressed as:

$$\tau_\chi = \alpha \frac{M_S w^2}{6\sigma\gamma\Delta} \tag{9}$$

where σ is the domain wall energy density at rest (see Ref. 4). The velocity saturates once the tilting is in its equilibrium state: see the black solid lines ($\alpha \sim 0.01$) in Fig. 2(a) and 2(c), which correspond to $\tau_\chi \sim 20$ ns. The 2D pinning and the wall tilting have little effect on the velocity for the short pulses because the time is not enough to develop sizeable amount of tilting.

Comparison to experimental results

The current density dependence of v_{END} for the ~5 μm wide wire (Supplementary Fig. 3(b)) is fitted with the 1D model that includes wall tilting and pinning. The model parameters are chosen based on the material parameters of film set B (see Supplementary Table 1). However, M_S used in the calculations is larger than that found in the experiments. M/V in Supplementary Table 1 underestimates the saturation magnetization since it includes information of magnetic dead layer. We thus use an intermediate value between M/V and the bulk M_S of $Co_{20}Fe_{60}B_{20}$ reported in the literature[16]. To account for the non-zero threshold current density, a one dimensional periodic pinning field[17] $H_{\mathrm{PIN}}(q) = \frac{1}{2M_S w t_{\mathrm{FM}}}\left(\frac{V_0 \pi}{q_0}\right) sin\left(\frac{\pi}{q_0} q\right)$ has been included in the model to fit the results: here $V_0 = 1.6 \times 10^{-11}$ erg and $q_0 = 7$nm are used. The fitting parameter is the DM exchange constant: the best fit gives $D = 0.24$ erg/cm^2, which is in agreement with that found from the fitting of

v_{END} vs. pulse amplitude (Fig. 1) using Eq. (1). Note that smaller V_0 and larger q_0 also provide reasonably good agreement for the results shown in Supplementary Fig. 3(b). To reproduce the pulse length dependence of v_{END} shown in Supplementary Fig. 4(a-c), however, small q_0 of ~5-10 nm is needed. Such small pinning periodicity is consistent with the amorphous structure of the CoFeB layer. With the D value obtained from the fitting, the equilibrium wall magnetization at rest is pointing close to the x axis, i.e. the wall forms a Neel-like structure[1,18].

The pulse length dependence of v_{END} is calculated using the 1D model with the parameters obtained above. The results are shown by the solid line (pinning is considered) and the dashed line (without pinning) in Supplementary Figs. 4(a-c). In agreement with the experiments, the calculated v_{END} increases with decreasing pulse length for short pulses ($t_{\mathrm{P}} \lesssim 20$ ns). However, the 1D model fails to reproduce the experimental results at longer pulses. Note that v_{END} vs. t_{P} is nearly identical for the tilted walls and the rigid walls (data not shown). In the 1D model, the wall tilting angle is underestimated due to the 1D nature of the pinning and consequently, the tilting has little effect on v_{END}. We find that the velocity reduction for longer pulses is only well reproduced when full micromagnetic simulations with realistic 2D disorder are considered.

Validity of the linearized equation of motion

The wall angle ψ changes from 0 (or π) to ±π/2 and vice versa when the current is tuned on and off. Thus the linearization process used to obtain the relaxation times (Eqs. (2) and (3)) needs justification. To study this, we have numerically calculated the domain wall velocity and extracted the relaxation times (τ_{A} and τ_{D}) using the solutions of the linearized model. Supplementary Figs. 6(a,b) show the instantaneous velocity as a function of time when a 100 ns long current pulse is applied. The parameters used are similar to those

described in Fig. 2 (with Gilbert damping α=0.05). For simplicity, we assume the tilt angle χ to be zero here. The acceleration and deceleration times are obtained by fitting the velocity vs. time using the following exponential function, the solution of the linearized equation (Supplementary Eq. (4)). With $v(t) = \frac{\partial q(t)}{\partial t}$, the solution takes the form:

$$v(t) = \begin{cases} v_1 \left[1 - \exp\left(-\frac{t}{\tau_{\mathrm{A}}}\right)\right] & \text{for } 0 \le t < t_{\mathrm{P}} \\ v_2 \exp\left(-\frac{t-t_{\mathrm{P}}}{\tau_{\mathrm{D}}}\right) & \text{for } t \ge t_{\mathrm{P}} \end{cases} \tag{10}$$

where $v_{1(2)}$ are the fitting parameters and t_{P} is the current pulse length. The boundary condition at $t = t_{\mathrm{P}}$ suggest that $v_1 = v_2$ (in the text, we use $v_1 = v_2 = v_{\mathrm{D}}$). Here, for the purpose of fitting, we use two different parameters $v_{1(2)}$ for the reason described below.

As the time variation of the wall angle is the main source of the relaxation effects, we have calculated and fitted ψ vs. time using similar exponential functions (i.e. replace $v_{1(2)}$ with $\psi_{1(2)}$): the calculated and fitted curves are shown in Supplementary Figs. 6(c,d). The left and right panels show calculation results using different current densities. Note that the equation of motion (Supplementary Eq. (4)) and its solution (Supplementary Eq. (10)), i.e. the exponential function, are valid only when the wall angle ψ is close to ±π/2 when the current is on and 0 (or π) when it is off. We therefore limit the fitting range to which the exponential function can be applied: to a time range in which deviation of ψ from its equilibrium value is less than ~20 deg. This is why we have to define the amplitudes of the exponential function ($v_{1(2)}$ and $\psi_{1(2)}$) separately when the current is on and off.

First, from the fitting, we obtain the saturation velocity (v_{D}) and the corresponding equilibrium wall angle (i.e. ψ_{eq}) when current is applied. v_{D} and ψ_{eq} are equivalent to, respectively, v_1 and ψ_1 in Supplementary Eq. (10). These quantities are plotted in Supplementary Figs. 6(e) and 6(f) using the solid symbols. (As a guide to the eye, the solid

line in Supplementary Fig. 6(e) shows the numerically calculated saturation velocity at the end of the current pulse (t=100 ns).). Supplementary Figs. 6(e) and 6(f) show that ψ_{eq} decreases with decreasing current density, resulting in a smaller velocity at lower current. In the parameter set used here, a considerably decrease in ψ_{eq} and v_{D} occurs when the current density is smaller than ~0.2×10^{8} A/cm^{2}.

The corresponding acceleration (τ_{A}) and deceleration (τ_{D}) times are shown in Supplementary Figs. 6(g) and 6(h). We show τ_{A} and τ_{D} obtained by fitting the velocity vs. time (black squares) and ψ vs. time (red circles) and compare those to the values calculated using Eqs. (2) and (3) (blue solid line). We find that the numerical calculations and the analytical solutions of the acceleration time τ_{A} are in good agreement even for small current densities at which ψ_{eq} is much smaller than π/2. These calculations show that the estimation of τ_{A} using Eq. (2) is valid at smaller current although its derivation assumes $\psi_{\mathrm{eq}} \approx \frac{\pi}{2}$.

The numerical calculations of the deceleration time (Supplementary Fig. 6(h), solid symbols) show that τ_{D} varies little with the current density. This is in good agreement with Eq. (3), which dictates that τ_{D} is constant against the current density. The numerical calculations of the deceleration time are ~10-20% smaller than the analytical estimate. From these results, we consider the expressions given in Eqs. (2) and (3) provide good estimates of the relaxation times.

The difficulty in fitting the relaxation process arises since the wall mass, or the relaxation time, continues to evolve during the transient processes. Under such condition, it is not appropriate to use a single relaxation time to describe the process. We have therefore limited the fitting range to estimate a relaxation time that more or less describes the equilibrium state (Supplementary Fig. 6). Ideally, to describe the relaxation process, one would need to use a relaxation time that is a weighted average of the processes involved

Numerical evaluation of the relaxation times

As the velocity or the wall angle cannot be fitted well with an exponential function with a constant relaxation time, we have computed the relaxation times numerically using the calculation results of the 1D model. To illustrate how the relaxation times are obtained numerically, we show in Supplementary Fig. 7(a) the temporal evolution of the wall velocity when a current pulse is applied. The results are similar to that presented in Fig. 2(a), solid line. The maximum velocity (v_{max}) and the velocity at the end of the pulse, i.e. the terminal velocity ($v(t_{\mathrm{P}})$) are defined schematically in Supplementary Fig. 7(a). The acceleration time (τ_{A}) is obtained by calculating the time needed to reach $\frac{2}{3}v_{\mathrm{max}}$ after the pulse is turned on. The deceleration time (τ_{D}) is estimated by time it takes to reach $\frac{1}{3}v(t_{\mathrm{P}})$ after the pulse is turned off. Although the relaxation times obtained in such a way quantitatively differ from those calculated using the linearized solutions (Eqs. (2) and (3)), the former provides a qualitative view of how the relaxation times depend on key material parameters.

The numerically calculated relaxation times are displayed in Supplementary Figs. 7(b-d) as a function of current density and in Supplementary Figs. 7(e-g) as a function of Gilbert damping, spin Hall angle and the DM exchange constant. The difference in τ_{A} and τ_{D} is apparent when the damping is small and when the DM exchange constant is small such that $\frac{\pi}{2}H_{\mathrm{DM}}$ approaches the domain wall anisotropy field (H_{K}). These results qualitatively support the relaxation times (Eqs .(2) and (3)) obtained using the linear approximation of the 1D model.

Supplementary Note 3

Full micromagnetic simulations

In order to further support the experimental observations and the 1D model calculations, full micromagnetic (µM) simulations have been performed by solving the Landau Lifshitz Gilbert equation augmented with the damping-like component of the spin Hall torque:

$$\frac{\partial \vec{m}}{\partial t} = -\gamma \vec{m} \times (\vec{H}_{\text{eff}} + \vec{H}_{\text{th}}) + \alpha \vec{m} \times \frac{\partial \vec{m}}{\partial t} + \gamma \frac{\hbar \theta_{\text{SH}} J(t)}{2e M_{\text{S}} L_{\text{z}}} \vec{m} \times (\vec{\sigma} \times \vec{m}) \quad (11)$$

where the effective field $\vec{H}_{\text{eff}}$ includes exchange, magnetostatic, magnetocristalline anisotropy (i.e. uniaxial perpendicular magnetic anisotropy) and Dzyaloshinskii-Moriya interactions. $\vec{H}_{\text{th}}$ is the thermal field and $\vec{\sigma} = \vec{u}_{\text{y}}$ is the polarization of the spin current (see Ref. [19] for numerical details) entering the magnetic layer. The material parameters are the same with those used for the 1D model: $M_{\text{S}} = 1100$ emu/cm^3, $A = 1.5 \times 10^{-6}$ erg/cm, $K_{\text{EFF}} \sim 3.2 \times 10^{6}$ erg/cm^3, $\theta_{\text{SH}} = -0.21$, $\alpha = 0.05$ and $D = 0.24$ erg/cm^2.

In order to take into account the effects of disorder due to imperfections and defects in a more realistic way than that of the 1D model, we assume the easy axis anisotropy direction is distributed among a length scale defined by a "grain" size. The grains vary in size taking an average diameter of $D_{\text{G}} = 30$ nm. The direction of the uniaxial anisotropy of each grain is mainly directed along the perpendicular direction (z-axis) but with a small in-plane component which is randomly generated over the grains. The maximum percentage of the in-plane component of the uniaxial anisotropy unit vector is varied from 10% to 15% ($0.10 \leq \varepsilon \leq 0.15$). In this work, we have computed the domain wall velocity as a function of pulse length for five different grain patterns (A-E) generated randomly and the average velocity are compared to the experimental results shown in Supplementary Fig. 4(a-c).

In order to evaluate the influence of the wall tilting, two strips with two different widths were studied numerically using 2D micromagnetic simulations: $w = 1536\,nm$ and $w = 4997\,nm$. Note that the latter is the same as that of the experimental wire studied in the main text. The strips are discretized using a finite difference scheme with cells composed of $3nm \times 3nm \times 1nm$: the thickness of the cell is the same with that of the CoFeB strip ($t_{\mathrm{FM}} = 1nm$). A micromagnetic study using the real dimensions of the experimental samples (~30-40 μm long wires) is not possible due to computer memory limitations. Therefore, the length of the strips considered in the modeling is $l = 12.3\,\mu m$. Similar to the experiments, two domain walls are placed in the strips and the current-driven motion of domain walls is evaluated. The quasi-static velocity v_{END} is estimated by dividing the total distance the domain wall traveled both during and after the current pulse application with the pulse length.

We first focus on the strip with a width of $w = 1536\,nm$ and study the effect of the degree of disorder (ε) on v_{END}. Supplementary Fig. 8 shows the simulated v_{END} as a function of the pulse length t_{P} for grain pattern A with three degrees of disorder: $\varepsilon = 0.10, 0.12, 0.15$. The current density is fixed to $J = 0.8 \times 10^8$ A/cm^2, a condition that corresponds to that of Supplementary Fig. 4(c). The t_{P} dependence of v_{END} is similar for the three degrees of disorder evaluated. In terms of quantitative agreement with the experiments (black solid circles in Supplementary Fig. 8) the best fit is found for the case with $\varepsilon = 0.12$. Based on this agreement, the degree of disorder is fixed to $\varepsilon = 0.12$ from hereafter.

The effects of grain pattern and temperature on v_{END} are presented in Supplementary Fig. 9. Similar results are obtained for different grain patterns and with different temperatures, i.e. zero and room temperature. The simulations are in good agreement with the experimental results. Snapshots of the magnetic contrast, before and after the current pulse application, are shown in Supplementary Figs. 9(d-f). For larger t_{P}, in contrast to the 1D model calculations, the tilting angle is non-zero even after the current pulse is turned off. In addition, we find that

the tilting angle during the current pulse is larger when the 2D pinning is introduced compared to that estimated using the 1D model. Thus for longer pulses, where the tilting becomes more significant, the velocity reduction is larger in the simulations and thus accounts for the gradual reduction of v_{END} with increasing t_{P}. For short pulses ($t_{\mathrm{P}} \lesssim 20$ ns), the inertia effect determines the enhancement in v_{END}, as predicted by the 1D model.

As the domain wall tilting scales with the strip width w, the strip width influences the time scale of domain wall tilting (see Supplementary Eq. 10) and therefore it can modify the domain wall velocity. In order to evaluate this effect a second micromagnetic study was carried out using the same strip width as in the experimental measurements ($w{\sim}5\ \mu m$). The results of these simulations are shown in Supplementary Fig. 10 in comparison to the experimental results. As it is clearly shown, the results for the wider strip are in very good agreement with the experimental data and they exhibit similar trend with the previous simulations for narrow wires in Supplementary Fig. 9. Agreement with experimental results is slightly better for simulations with the wider wires. Note that the increase in the velocity at shorter pulses is a little more abrupt for the narrower wire (Supplementary Fig. 9) compared to that of the wider wire (Supplementary Fig. 10), which is due to the wire width dependent time scale of domain wall tilting.

These micromagnetic simulations corroborate the experimental results and the interpretation based on the 1D model discussed in the main text. The 1D model description is valid for short current pulses with $t_{\mathrm{P}} \lesssim 10-20$ ns. However, as the pulse length increases, the model fails to provide a quantitative agreement with the experimental results. The reasons behind this are described as follows. In the framework of the 1D model, the pinning is introduced as a one dimensional space-dependent effective field defined by a given energy barrier and a period. This pinning field is purely 1D (only depends on the x coordinate), and therefore it cannot capture the 2D pinning present in real samples. As t_{P} increases, the

domain wall tilting increases, however its degree is larger when a 2D pinning is assumed. Since the velocity becomes smaller as the tilting increases, the velocity reduction is larger in the simulations (compared to the 1D model calculations) due to the 2D pinning that gives rise to larger tilting.

Supplementary References

1 Torrejon, J., Kim, J., Sinha, J., Mitani, S., Hayashi, M., Yamanouchi, M. & Ohno, H. *Interface control of the magnetic chirality in CoFeB/MgO heterostructures with heavy-metal underlayers*. Nat. Commun. **5**, 4655 (2014).

2 Liu, J., Ohkubo, T., Mitani, S., Hono, K. & Hayashi, M. *Correlation between the spin Hall angle and the structural phases of early 5d transition metals*. Appl. Phys. Lett. **107**, 232408 (2015).

3 Ryu, K. S., Thomas, L., Yang, S. H. & Parkin, S. S. P. *Current Induced Tilting of Domain Walls in High Velocity Motion along Perpendicularly Magnetized Micron-Sized Co/Ni/Co Racetracks*. Appl. Phys. Express **5**, 093006 (2012).

4 Boulle, O., Rohart, S., Buda-Prejbeanu, L. D., Jue, E., Miron, I. M., Pizzini, S., Vogel, J., Gaudin, G. & Thiaville, A. *Domain Wall Tilting in the Presence of the Dzyaloshinskii-Moriya Interaction in Out-of-Plane Magnetized Magnetic Nanotracks*. Phys. Rev. Lett. **111**, 217203 (2013).

5 Martinez, E., Emori, S., Perez, N., Torres, L. & Beach, G. S. D. *Current-driven dynamics of Dzyaloshinskii domain walls in the presence of in-plane fields: Full micromagnetic and one-dimensional analysis*. J. Appl. Phys. **115**, 213909 (2014).

6 Emori, S., Martinez, E., Lee, K.-J., Lee, H.-W., Bauer, U., Ahn, S.-M., Agrawal, P., Bono, D. C. & Beach, G. S. D. *Spin Hall torque magnetometry of Dzyaloshinskii domain walls*. Phys. Rev. B **90**, 184427 (2014).

7 Thiaville, A., Rohart, S., Jue, E., Cros, V. & Fert, A. *Dynamics of Dzyaloshinskii domain walls in ultrathin magnetic films*. Europhys. Lett. **100**, 57002 (2012).

8 Ryu, K.-S., Thomas, L., Yang, S.-H. & Parkin, S. *Chiral spin torque at magnetic domain walls*. Nat. Nanotechnol. **8**, 527-533 (2013).

9 Emori, S., Bauer, U., Ahn, S.-M., Martinez, E. & Beach, G. S. D. *Current-driven dynamics of chiral ferromagnetic domain walls*. Nat. Mater. **12**, 611-616 (2013).

10 Martinez, E. & Alejos, O. *Coupled Dzyaloshinskii walls and their current-induced dynamics by the spin Hall effect*. J. Appl. Phys. **116**, 023909 (2014).

11 Tarasenko, S. V., Stankiewicz, A., Tarasenko, V. V. & Ferre, J. *Bloch wall dynamics in ultrathin ferromagnetic films*. J. Magn. Magn. Mater. **189**, 19-24 (1998).

12 Liu, L. Q., Moriyama, T., Ralph, D. C. & Buhrman, R. A. *Spin-Torque Ferromagnetic Resonance Induced by the Spin Hall Effect*. Phys. Rev. Lett. **106**, 036601 (2011).

13 Liu, L., Pai, C.-F., Li, Y., Tseng, H. W., Ralph, D. C. & Buhrman, R. A. *Spin-Torque Switching with the Giant Spin Hall Effect of Tantalum*. Science **336**, 555-558 (2012).

14 Fukami, S., Suzuki, T., Nakatani, Y., Ishiwata, N., Yamanouchi, M., Ikeda, S., Kasai, N. & Ohno, H. *Current-induced domain wall motion in perpendicularly magnetized CoFeB nanowire*. Appl. Phys. Lett. **98**, 082504 (2011).

15 Thomas, L., Moriya, R., Rettner, C. & Parkin, S. S. P. *Dynamics of Magnetic Domain Walls Under Their Own Inertia*. Science **330**, 1810-1813 (2010).

16 Bozorth, R. M. *Ferromagnetism*. (Wiley-IEEE Press, 1993).

17 Martinez, E. *The stochastic nature of the domain wall motion along high perpendicular anisotropy strips with surface roughness*. J. Phys.-Condes. Matter **24**, 024206 (2012).

18 Gross, I., Martínez, L. J., Tetienne, J. P., Hingant, T., Roch, J. F., Garcia, K., Soucaille, R., Adam, J. P., Kim, J. V., Rohart, S., Thiaville, A., Torrejon, J., Hayashi, M. & Jacques, V. *Direct measurement of interfacial Dzyaloshinskii-Moriya interaction in X|CoFeB|MgO heterostructures with a scanning NV magnetometer (X=Ta, TaN, and W)*. Phys. Rev. B **94**, 064413 (2016).

19 Vansteenkiste, A., Leliaert, J., Dvornik, M., Helsen, M., Garcia-Sanchez, F. & Van Waeyenberge, B. *The design and verification of MuMax3*. AIP Advances **4**, 107133 (2014).
